# Health-related quality of life in patients with atrial fibrillation: The role of symptoms, comorbidities, and the type of atrial fibrillation

**DOI:** 10.1371/journal.pone.0226730

**Published:** 2019-12-23

**Authors:** Fabienne Witassek, Anne Springer, Luise Adam, Stefanie Aeschbacher, Jürg H. Beer, Steffen Blum, Leo H. Bonati, David Conen, Richard Kobza, Michael Kühne, Giorgio Moschovitis, Stefan Osswald, Nicolas Rodondi, Christian Sticherling, Thomas Szucs, Matthias Schwenkglenks, for the Swiss-AF study investigators

**Affiliations:** 1 Epidemiology, Biostatistics and Prevention Institute, University of Zurich, Zurich, Switzerland; 2 Cardiovascular Research Institute Basel, University Hospital Basel, Basel, Switzerland; 3 Division of Cardiology, Department of Medicine, University Hospital Basel, Basel, Switzerland; 4 Department of General Internal Medicine, Inselspital, Bern University Hospital, University of Bern, Bern, Switzerland; 5 Department of Medicine, Cantonal Hospital of Baden, Baden, and Molecular Cardiology, University Hospital of Zurich, Zurich, Switzerland; 6 Neurology Division and Stroke Centre, Department of Clinical Research, University Hospital Basel, Basel, Switzerland; 7 Population Health Research Institute, McMaster University, Hamilton, Canada; 8 Department of Cardiology, Luzerner Kantonsspital, Lucerne, Switzerland; 9 Division of Cardiology, Ente Ospedaliero Cantonale, Ospedale Regionale di Lugano, Ticino, Lugano, Switzerland; 10 Institute of Primary Health Care (BIHAM), University of Bern, Berne, Switzerland; 11 Institute of Pharmaceutical Medicine (ECPM), University of Basel, Basel, Switzerland; Policlinico Casilino, ITALY

## Abstract

**Aims:**

This study aimed to analyse health related quality of life (HRQoL) for patients with different atrial fibrillation (AF) types and to identify patient characteristics, symptoms and comorbidities that influence HRQoL.

**Methods:**

We used baseline data from the Swiss Atrial Fibrillation (Swiss-AF) study, a prospective multicentre observational cohort study conducted in 13 clinical centres in Switzerland. Between April 2014 and August 2017, 2415 AF patients were recruited. Patients were included in this analysis if they had baseline HRQoL data as assessed with EQ-5D-based utilities and visual analogue scale (VAS) scores. Patient characteristics and HRQoL were described stratified by AF type. The impact of symptoms, comorbidities and socio-economic factors on HRQoL was analysed using multivariable regression analysis.

**Results:**

Based on 2412 patients with available baseline HRQoL data, the lowest unadjusted mean HRQoL was found in patients with permanent AF regardless of whether measured with utilities (paroxysmal: 0.83, persistent: 0.84, permanent: 0.80, p<0.001) or VAS score (paroxysmal: 73.6, persistent: 72.8, permanent: 69.2, p<0.001). In multivariable analysis of utilities and VAS scores, higher European Heart Rhythm Association (EHRA) score, recurrent falls and several comorbidities showed a strong negative impact on HRQoL while AF type was no longer associated with HRQoL.

**Conclusions:**

Multiple factors turned out to influence HRQoL in AF patients. After controlling for several comorbidities, the EHRA score was one of the strongest predictors independent of AF type. The results may be valuable for better patient assessment and provide a reference point for further QoL and health economic analyses in AF populations.

## Introduction

Atrial fibrillation (AF), the most common cardiac arrhythmia with an estimated prevalence of 2% in the general adult population of Europe [1], is associated with a broad range of symptoms such as palpitations, dyspnoea, chest tightness, lethargy, sleeping difficulties, and psychosocial distress [2]. In addition to the burden of the disease itself, patients with AF face an increased risk for major complications such as heart failure, cognitive impairment, and stroke [3]. All these factors may, depending on their grade of manifestation, impact the health-related quality of life (HRQoL) of AF patients.

Previous studies investigating the impact of AF on HRQoL found poorer HRQoL in AF patients compared to the general population [4–6]. However, other studies demonstrated that comorbid conditions were more strongly related with HRQoL than the clinical manifestations of AF itself [7, 8], and that HRQoL was mainly impaired in newly diagnosed patients and rose to a normal level with standard treatment [7]. A further study showed that HRQoL was significantly impacted by AF type and symptoms in addition to comorbidities, regardless of disease duration [9].

AF is classified as paroxysmal (i.e., self-terminating AF lasting <7 days that does not require cardioversion), persistent (i.e., AF sustained ≥7 days and/or requiring cardioversion) or permanent (i.e., cardioversion has failed or not been attempted) [10]. Patient characteristics usually differ by AF type, with permanent AF patients being older and showing more comorbidities [11]. AF symptom burden, on the other hand, is often higher in paroxysmal or persistent AF according to previous studies [2, 12]. HRQoL may thus differ depending on the impact and strength of symptoms and comorbidities. Whether AF type itself plays an independent role in HRQoL is not clear. The few available studies of differences in HRQoL across AF types found either no independent difference [13] or lower HRQoL for paroxysmal and permanent AF [9].

Given these inconsistent findings, we aimed to investigate whether and how HRQoL varies between AF types, to examine which patient characteristics, symptoms and comorbidities are mainly influencing HRQoL, and to investigate whether the influence of certain symptoms or comorbidities on HRQoL is more pronounced within specific AF types.

## Materials and methods

### Data source

Swiss-AF is a prospective multicentre observational cohort study conducted in 13 clinical centres in Switzerland with the aim to provide new insights on structural and functional brain damage in patients with AF and to investigate other AF-related complications and burden, collecting a large variety of clinical, genetic, phenotypic and health economic data [14, 15].

Recruitment started in April 2014 and was completed in August 2017. Patients were enrolled if they were at least 65 years old. An additional subgroup of 200 patients aged between 45–65 years was enrolled as an additional aim of the cohort was to assess socio-economic aspects of AF in the working population. Participants had to have documented paroxysmal AF (at least twice within the last 60 months), persistent AF (documented within the last 60 months by ECG or rhythm monitoring devices) or permanent AF. The detailed study set-up has previously been described [14]. The study protocol has been approved by the Ethics Committees of Northwest and Central Switzerland (EKNZ), and an informed written consent was obtained from each participant.

Patients were included in this analysis if they had baseline data on HRQoL assessed with the three-level version of the EQ-5D (EQ-5D-3L). The EQ-5D-3L is a standardized instrument to assess generic HRQoL and contains questions on five dimensions: mobility, self-care, usual activities, pain/discomfort, and anxiety/depression. For each of the five dimension, respondents are offered three response categories (no problems, some problems, extreme problems), leading to 243 possible health states [16, 17]. These health states are then converted into index based values (utilities) ranging from 0 to 1 by applying a country-specific valuation algorithm. As no Swiss value set is available, we used the European Value set (VAS validated) to calculate utilities [18].

Additionally, the instrument includes a visual analogue scale (VAS), on which the patient is asked to score his/her current health state between 0 (worst imaginable health state) and 100 (best imaginable health state).

In addition to single symptoms, such as palpitations, fatigue, or dizziness, we integrated the European Heart Rhythm Association (EHRA) classification score into the analysis. The EHRA score describes the severity of AF-related symptoms, specifically during the time when the patient feels to be in the arrhythmia, and distinguishes four classes: “No symptoms” (I), “mild symptoms” (II), “severe symptoms” (III), and “disabling symptoms” (IV) [19].

EHRA scores, symptoms, comorbidities and the questionnaire part of the EQ-5D-3L instrument were assessed by the study personnel during patient interviews. If relevant, medical records were additionally consulted. The VAS part of the EQ-5D-3L instrument was completed directly by the patients if possible.

### Statistical analysis

Baseline characteristics, symptoms, EHRA score and HRQoL results (i.e., EQ-5D utilities and VAS scores) are presented stratified by AF type. Discrete variables are reported as frequencies and percentages and continuous variables as means and standard deviations. To gain an initial understanding of differences between AF types standard univariable tests were used (Pearson chi-square test or the Fisher’s exact test, Analysis of Variance (ANOVA)). To visualize the relative importance of comorbidities according to AF type, a grouped bar chart of proportional occurrence is shown. Additionally, we analysed how the observed utilities or VAS scores are associated with the EHRA score. Boxplots were used to visually describe the association between the EHRA score and HRQoL measurements. The Kruskal-Wallis test and Spearman’s correlation coefficients were used to test for HRQoL differences between EHRA classes.

Covariate influences on HRQoL were subsequently assessed using linear mixed-effects models with random intercepts for centre to take into account possible effects related to the different study centres. Possible covariates were selected based on literature review and clinical experience. To pre-assess candidate covariates representing symptoms, comorbidities, AF type, ECG at study visit and treatments as antiarrhythmic drugs, devices and previous pulmonary vein isolation (PVI) procedures, standard univariable analyses of associations with utilities or VAS scores were performed. Covariates were considered for a multivariable model if they showed a p-value < 0.2 in the univariable analysis. In the multivariable analysis, covariates with a p-value higher than 0.2 were excluded from the model. Collinearity was not observed during analysis. Clinical observations by the study team led to a notion that the impact of comorbidities on HRQoL might be more pronounced in permanent AF patients than in paroxysmal or persistent AF patients while symptoms might be stronger predictors in patients with paroxysmal or persistent AF. In order to assess this assumption, multiplicative interactions between AF type and symptoms or comorbidities were tested and included in the final model if they were statistically significant and lowered the Bayesian information criterion (BIC). The BIC is a criterion for model selection and implies a penalisation for over-complexity. A decrease in BIC indicates an improvement in model fit and better predictive ability [20]. To account for an expected ceiling effect at the value 1 for the utility values, we complemented the linear regression with a Tobit regression, often used to modelling censored variables in econometrics research [21].

All analyses were performed using STATA 13.1 and a p-value <0.05 was considered as threshold for statistical significance.

## Results

### Patients and symptom burden

**Fig 1:**
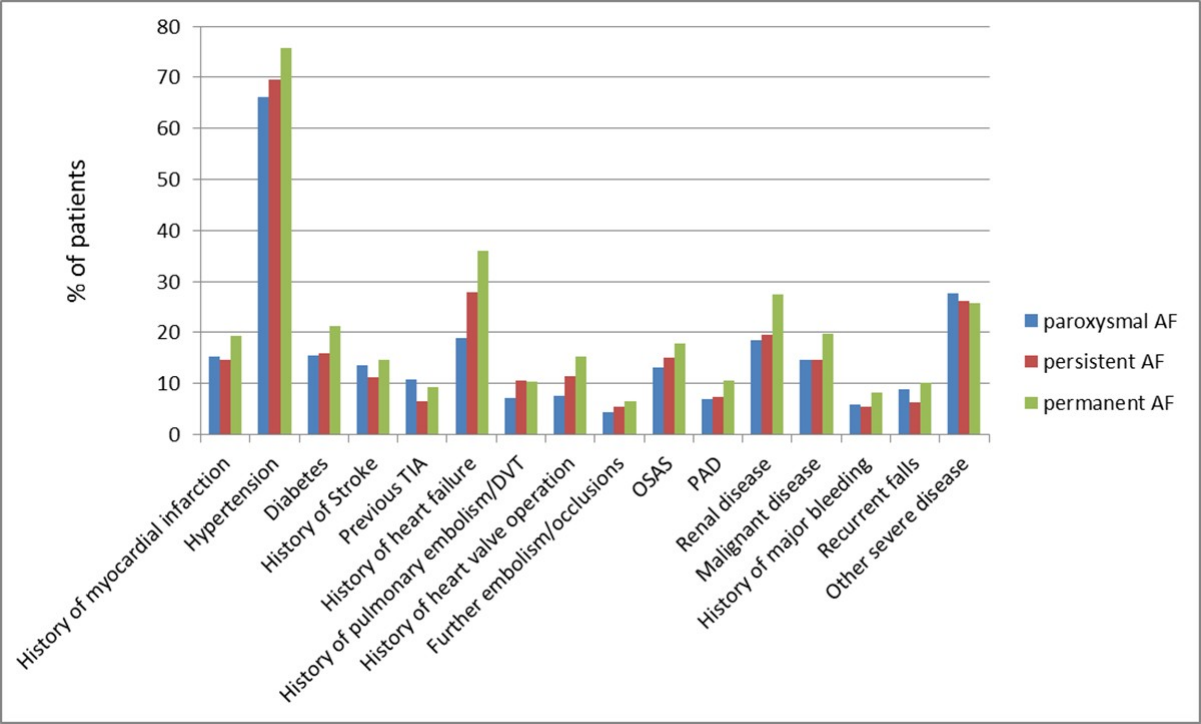
Frequencies of comorbidities according to AF type. TIA, transient ischemic attack; DVT, deep vein thrombosis; OSAS, obstructive sleep apnoea syndrome; PAD, peripheral artery disease.

Of the 2415 patients enrolled in the study, 2412 (99.9%) completed the baseline EQ-5D and were included in this analysis. The mean age was 73.2 years (IQR 68; 79) and 72.6% of the patients were male. AF type at baseline was paroxysmal for 1079 (44.7%) patients, persistent for 709 (29.4%) and permanent for 624 (25.9%). Patients with permanent AF were older and had higher rates of comorbidities as compared to patients with paroxysmal or persistent AF (Table 1 and S1 Table). For all three AF types, the most frequent comorbidities were hypertension and heart failure (Fig 1). The patients with persistent AF showed the highest use of antiarrhythmic drugs at baseline. The prevalence of previous PVI was similar in paroxysmal and persistent AF and significantly lower in permanent AF.

**Table 1:** Baseline characteristics according to AF type. BMI, body mass index; NOACs, new oral anticoagulants; MoCA, Montreal Cognitive Assessment.

		Paroxysmal	Persistent	Permanent	p
**N (%)**		1079 (44.7)	709 (29.4)	624 (25.9)	
**Age**	mean (SD)	72.5 (8.6)	71.8 (8.5)	76.3 (7.4)	**<0.001**
**Age groups**					**<0.001**
<65	n/N (%)	143/1079 (13.3)	109/709 (15.4)	34/624 (5.4)	
65-<75	n/N (%)	480/1079 (44.5)	329/709 (46.4)	216/624 (34.6)	
75-<85	n/N (%)	389/1079 (36.1)	235/709 (33.1)	291/624 (46.6)	
> = 85	n/N (%)	67/1079 (6.2)	36/709 (5.1)	83/624 (13.3)	
**Male**	n/N (%)	737/1079 (68.3)	531/709 (74.9)	484/624 (77.6)	**<0.001**
**BMI**	mean (SD)	27.3 (4.9)	27.9 (4.7)	28.1 (4.6)	**0.001**
**Time since first diagnosis (years)**	mean (SD) range	4.9 (5.9) 0–52.8	5.1 (7.3) 0–55.7	9.6 (9.5) 0–63.8	**<0.001**
**Antiarrhythmics at baseline**	n/N (%)	272/1079 (25.2)	228/708 (32.2)	164/623 (26.3)	**0.004**
**Oral Anticoagulation at baseline**					**<0.001**
NOACs	n/N (%)	614/1078 (57.0)	416/709 (58.7)	199/624 (31.9)	
Vitamin K antagonists	n/N (%)	318/1078 (29.5)	245/709 (34.6)	388/624 (62.2)	
none	n/N (%)	146/1078 (13.5)	48/709 (6.8)	37/624 (5.9)	
**History of PVI**	n/N (%)	270/1079 (25.0)	177/709 (25.0)	41/624 (6.6)	**<0.001**
**Device** (PM, CRT, CRT-ICD, ICD, loop recorder)	n/N (%)	209/1079 (19.4)	118/709 (16.6)	153/624 (24.5)	**0.001**
**MoCA Score**	mean (SD) range	25.1 (3.2) 10–30	25.1 (3.2) 9–30	24.2 (3.4) 7–30	**<0.001**
**AF or Flutter at study visit (ECG)**	n/N (%)	180/1071 (16.8)	292/705 (41.4)	588/624 (94.2)	**<0.001**

AF-related symptoms were more frequent in paroxysmal and persistent AF than in permanent AF. Consistent with this observation, symptom severity, as measured by the EHRA score, was higher for paroxysmal and persistent AF. Across all three AF types, more than 50% of the patients were in EHRA class I and hence free of AF-related symptoms (Table 2).

**Table 2:** Symptoms and EHRA Score according to AF type. EHRA, European Heart Rhythm Association.

		Paroxysmal	Persistent	Permanent	p
**Symptoms related to AF**					
Palpation	n/N (%)	511/1076 (47.5)	224/709 (31.6)	134/624 (21.5)	**<0.001**
Dizziness	n/N (%)	194/1076 (18.0)	94/709 (13.3)	53/624 (8.5)	**<0.001**
Chest pain	n/N (%)	134/1076 (12.5)	50/709 (7.1)	52/624 (8.3)	**<0.001**
Exercise intolerance	n/N (%)	245/1076 (22.8)	206/709 (29.1)	87/624 (13.9)	**<0.001**
Dyspnea	n/N (%)	239/1076 (22.2)	202/709 (28.5)	148/624 (23.7)	**0.009**
Fatigue	n/N (%)	183/1076(17.0)	133/709 (18.8)	70/624 (11.2)	**<0.001**
Syncopes	n/N (%)	47/1076 (4.4)	16/709 (2.3)	15/624 (2.4)	**0.019**
None	n/N (%)	312/1076(29.0)	270/709 (38.1)	336/624 (53.8)	**<0.001**
**EHRA Score**					**0.001**
I	n/N (%)	606/1078 (56.2)	390/709 (55.0)	410/624 (65.7)	
II	n/N (%)	365/1078 (33.8)	237/709 (33.4)	170/624 (27.2)	
III	n/N (%)	83/1078 (7.7)	68/709 (9.6)	37/624 (5.9)	
IV	n/N (%)	24/1078 (2.2)	14/709 (2.0)	7/624 (1.1)	
>II	n/N (%)	107/1078 (9.9)	82/709 (11.6)	44/624 (7.1)	**0.019**

### Quality of life by AF type

The lowest unadjusted average HRQoL was found in the permanent AF group, regardless of whether measured with EQ-5D questionnaire results converted to utilities (paroxysmal: 0.83, persistent: 0.84, permanent: 0.80, p<0.001) or the VAS score (paroxysmal: 73.60, persistent: 72.78, permanent: 69.17, p<0.001). Women had lower utilities and VAS scores than men, across all three AF types (Table 3).

**Table 3:** EQ-5D-EU-Utilities and VAS Scores according to AF type.

		All	Paroxysmal	Persistent	Permanent	p
**All:**						
*Health Utility EU*	Mean (SD)	0.82 (0.17)	0.83 (0.17)	0.84 (0.17)	0.80 (0.18)	**<0.001**
	Range		0.12–1.00	0.04–1.00	0.00–1.00	
*VAS Score*	Mean (SD)	72.20 (17.48)	73.60 (17.24)	72.78 (17.74)	69.17 (17.27)	**<0.001**
	Range		3.00–100.00	0.00–100.00	5.00–100.00	
**Male:**						
*Health Utility EU*	Mean (SD)		0.85 (0.17)**	0.85 (0.16)*	0.82 (0.17)**	**<0.001**
	Range		0.14–1.00	0.24–1.00	0.00–1.00	
*VAS Score*	Mean (SD)		74.86 (17.12)**	73.36 (17.70)	69.99 (17.02)*	**<0.001**
	Range		3.00–100.00	0.00–100.00	25.00–100.00	
**Female:**						
*Health Utility EU*	Mean (SD)		0.78 (0.18)**	0.82 (0.19)*	0.76 (0.19)**	**0.016**
	Range		0.12–1.00	0.04–1.00	0.08–1.00	
*VAS Score*	Mean (SD)		70.81 (17.19)**	71.03 (17.77)	66.32 (17.88)*	**0.024**
	Range		8.00–100.00	0.00–100.00	5.00–100.00	

**p<0.001 and

*p<0.05 between male and female.

After correcting for age, gender, comorbidities, EHRA score, and education, AF type was no longer associated with utilities (joint p-value = 0.054) (Table 4) or VAS scores (joint p-value = 0.634) (Table 5). However, based on the VAS scores, the presence of AF or atrial flutter at the baseline visit showed an independent, significant impact on HRQoL (-1.776, p = 0.045).

**Table 4:** Multivariable regression analysis: Predictors of utility in AF patients. Joint p values: AF type p = 0.054, age p<0.001, EHRA Score p = 0.001, Education level p = 0.004. Study centre was included as a random effect variable in the model. OSAS, obstructive sleep apnoea syndrome; PAD, peripheral artery disease; DVT, deep vein thrombosis; EHRA, European Heart Rhythm Association.

	EQ-5D utility			
	Coef.	p-value	95% CI	
**AF type (paroxysmal as reference)**				
persistent	0.008	0.340	-0.008	0.023
permanent	-0.014	0.092	-0.031	0.002
**Age groups (<65 as reference)**				
65-<75	0.012	0.284	-0.009	0.034
75-<85	-0.012	0.288	-0.035	0.010
> = 85	-0.049	**0.002**	-0.082	-0.018
Female	-0.039	**<0.001**	-0.054	-0.023
Dizziness	-0.022	**0.027**	-0.041	-0.002
Chest pain	-0.022	0.057	-0.044	0.001
Fatigue	-0.016	0.086	-0.035	0.002
Recurrent falls	-0.065	**<0.001**	-0.089	-0.041
Malignant disease	-0.021	**0.019**	-0.039	-0.003
OSAS	-0.028	**0.003**	-0.046	-0.009
PAD	-0.034	**0.006**	-0.058	-0.009
Hypertension	-0.024	**0.001**	-0.039	-0.009
Diabetes	-0.031	**0.001**	-0.050	-0.013
Heart failure	-0.012	0.137	-0.028	-0.004
Renal insufficiency	-0.026	**0.002**	-0.044	-0.009
History of pulmonary embolism/DVT	-0.052	**<0.001**	-0.075	-0.030
History of stroke	-0.017	0.082	-0.036	0.002
**EHRA Score (1 as reference)**				
EHRA Score 2	-0.014	0.071	-0.029	0.001
EHRA Score 3	-0.042	**0.002**	-0.068	-0.016
EHRA Score 4	-0.069	**0.006**	-0.118	-0.019
**Educational level (basic as reference)**				
middle	0.016	0.151	-0.006	0.037
advanced	0.034	**0.003**	0.011	0.056
Constant	0.930	<0.001	0.892	0.970

**Table 5:** Multivariable regression analysis: Predictors of the VAS score in AF patients. Joint p values: AF type p = 0.634, age p<0.001, EHRA Score p<0.001, Education level p = 0.010. Study centre was included as random effect in the model. OSAS, obstructive sleep apnoea syndrome; PAD, peripheral artery disease; DVT, deep vein thrombosis; TIA, transient ischemic attack; EHRA, European Heart Rhythm Association; PVI, pulmonary vein isolation.

	VAS Score			
	Coef.	p-value	95% CI	
**AF type (paroxysmal as reference)**				
persistent	-0.601	0.464	-2.208	1.006
permanent	-0.933	0.380	-3.016	1.151
**Age groups (<65 as reference)**				
65-<75	1.922	0.084	-0.261	4.106
75-<85	-0.428	0.719	-2.763	1.907
> = 85	-2.555	0.121	-5.787	0.676
Female	-2.527	**0.002**	-4.118	-0.936
Chest pain	-4.009	**<0.001**	-6.220	-1.799
Fatigue	-1.664	0.078	-3.518	0.189
Recurrent falls	-3.467	**0.005**	-5.865	-1.068
Malignant disease	-5.243	**<0.001**	-7.012	-3.474
OSAS	-2.848	**0.003**	-4.710	-0.986
PAD	-3.104	**0.013**	-5.554	-0.655
Hypertension	-1.999	**0.007**	-3.463	-0.536
Diabetes	-3.039	**0.001**	-4.850	-1.227
Heart failure	-3.297	**<0.001**	-4.899	-1.694
Renal insufficiency	-3.528	**<0.001**	-5.231	-1.825
History of pulmonary embolism/DVT	-1.942	0.092	-4.202	0.319
History of myocardial infarction	-3.510	**<0.001**	-5.335	-1.686
History of stroke	-3.865	**<0.001**	-5.789	-1.940
AF/Flutter at study visit	-1.776	**0.045**	-3.515	-0.037
Previous PVI	1.770	0.052	-0.012	3.552
**EHRA Score (1 as reference)**				
EHRA Score 2	-2.594	**0.001**	-4.081	-1.107
EHRA Score 3	-6.557	**<0.001**	-9.122	-3.993
EHRA Score 4	-4.872	**0.050**	-9.737	-0.006
**Educational level (basic as reference)**				
middle	3.146	**0.004**	1.012	5.280
advanced	3.304	**0.004**	1.056	5.552
Constant	82.099	<0.001	78.177	86.019

### The EHRA score as a marker of HRQoL

**Fig 2:**
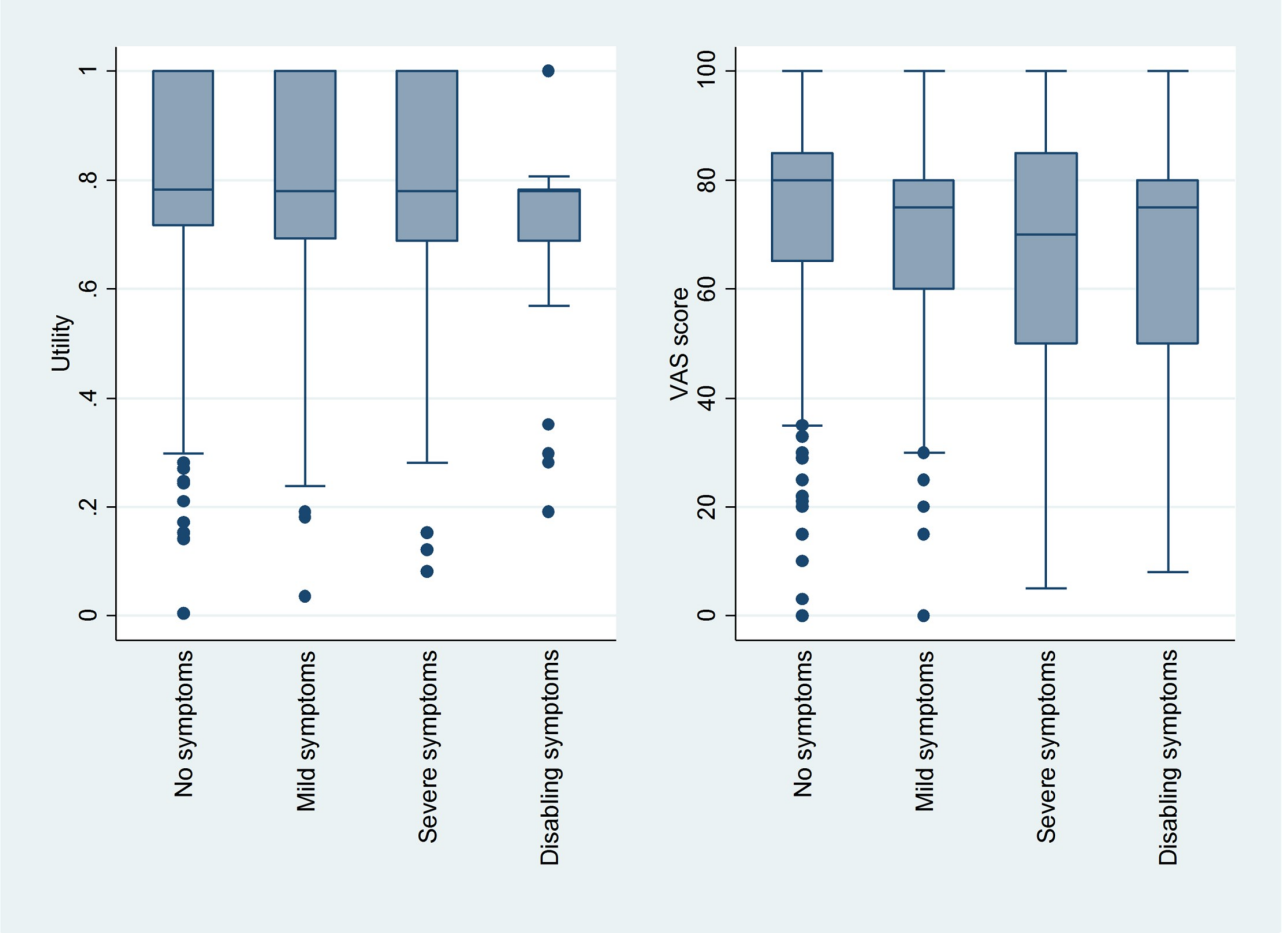
Boxplots of utility and VAS score according to the EHRA class.

Fig 2 shows unadjusted utilities and VAS scores by EHRA score across all patients. Between-group differences according to the Kruskal-Wallis test were significant for both (p<0.001). Spearman’s correlation coefficients also indicated decreased HRQoL with increasing EHRA score (utilities: r = -0.13, p<0.001; VAS score: r = -0.12, p<0.001). However, differences between medians were more pronounced in the VAS scores. There was no difference between EHRA classes 3 and 4, with EHRA class 4 represented only by 45 patients.

### The impact of symptoms and comorbidities on HRQoL

Multivariable analysis additionally indicated that several symptoms and comorbidities were independently associated with HRQoL. The strongest independent predictors of lower utility were higher EHRA class (-0.042 for class 3; -0.069 for EHRA class 4, joint p-value = 0.001), recurrent falls (-0.065, p<0.001) and history of pulmonary embolism/deep vein thrombosis (DVT) (-0.052, p<0.001). Further significant negative predictors were presence of malignant disease, sleep apnoea, peripheral artery disease (PAD), hypertension, diabetes and renal insufficiency. Lower education and symptoms of dizziness were also associated with lower utility. Effects of chest pain and fatigue trended towards lower utility (Table 4). If the EHRA score was tentatively excluded from the model these effects became significant. The ECG during the baseline visit and treatment variables representing the use of antiarrhythmic drugs, history of PVI and implanted device showed no impact and were excluded from the final model.

The strongest independent predictor of lower VAS scores was again higher EHRA class (-2.594 for class 2; -6.557 for class 3; joint p-value<0.001). Other than in the model of utility, history of pulmonary embolism/DVT played only a minor role but history of malignant disease (-5.243, p<0.001) was one of the strongest predictors for HRQoL. Further significant predictors of lower VAS scores were chest pain, recurrent falls, sleep apnoea, PAD, hypertension, diabetes, heart failure, renal insufficiency, myocardial infarction, stroke and lower education (Table 5). In this model also, use of antiarrhythmic drugs and implanted device showed no impact on HRQoL and were thus excluded. AF or atrial flutter during study visit showed a significant negative impact on HRQoL while previous PVI did not yield any effect in the final model.

Testing of interaction effects between AF type and symptoms and comorbidities, respectively, indicated that sleep apnoea had a negative effect on utilities in paroxysmal and permanent AF but not in persistent AF (p for interaction = 0.049). The negative effect of chest pain seemed to be more pronounced in persistent AF as compared to the other two AF types when measuring HRQoL with the VAS Score (p for interaction = 0.015). Also, PAD showed a negative effect on VAS scores in paroxysmal and persistent AF but a positive effect in permanent AF (p for interaction = 0.012). According to the BIC criterion, the interaction terms did not improve model fit and predictive ability. Hence, they were not included in the main models represented in Tables 4 and 5 but shown in [Online Appendix].

The results of the Tobit regressions were fully consistent with those of the linear regression (details not shown).

## Discussion

This cross-sectional analysis of 2412 AF patients indicated that HRQoL was mainly influenced by symptom severity and comorbidities, but not independently by the type of AF. We had expected that type of AF might impact QoL not only due to immediate symptom burden but also due to factors such as the frequency of symptoms, related fear of symptoms occurring, or habituation in case of permanent AF. However, or results did not show this. VAS scores were negatively influenced if the patient had AF or atrial flutter in the baseline ECG during the study visit.

We had expected that the impact of comorbidities on HRQoL might be more pronounced in permanent AF patients than in paroxysmal or persistent AF patients and that on the other hand, symptom severity might be a stronger predictor in patients with paroxysmal or persistent AF. However, after testing for interactions we found only interactions with no obvious clinical interpretation and could therefore not confirm such a relationship.

**Fig 3:**
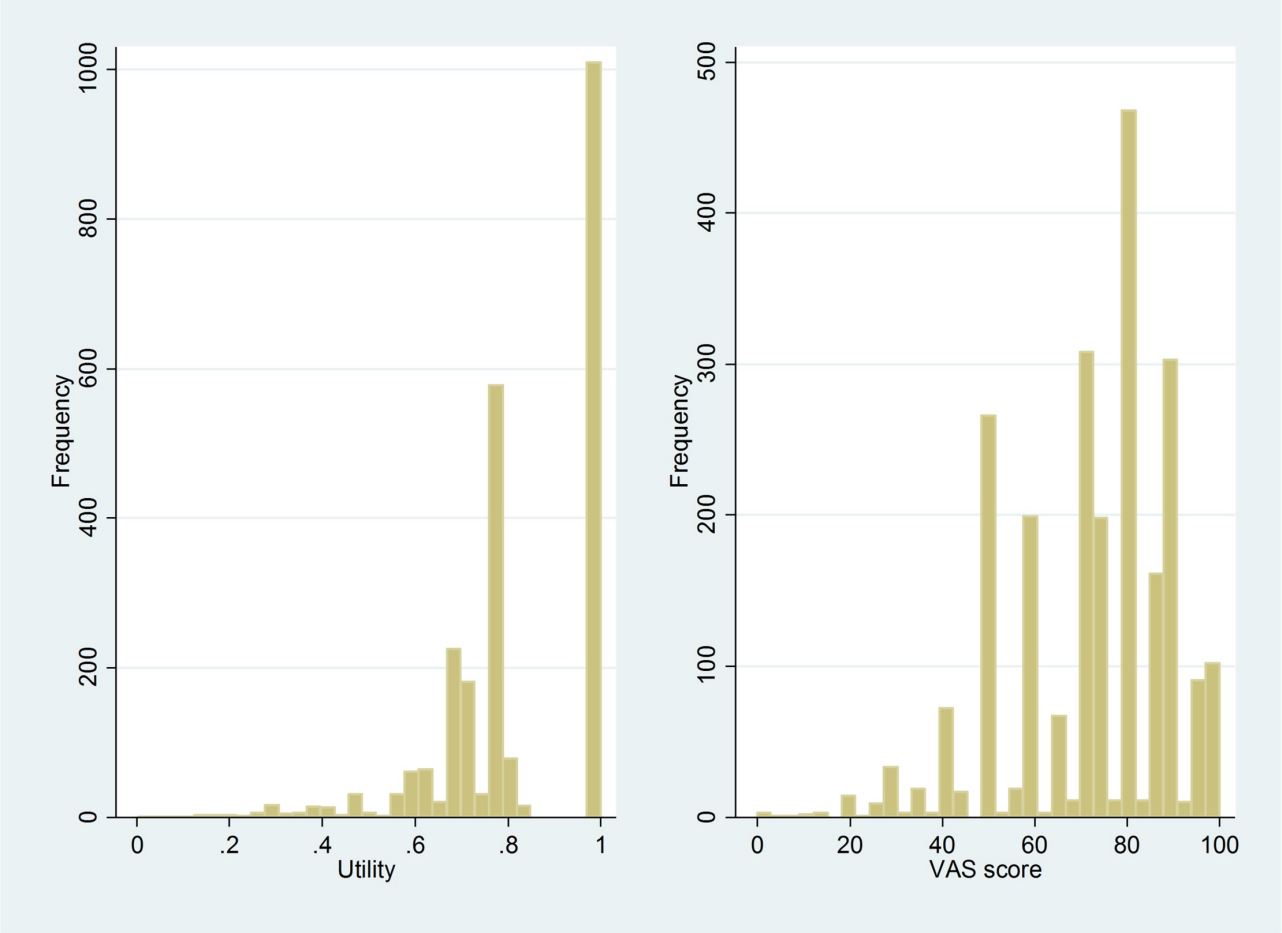
Distribution of utility and VAS score in the study population.

In general, when analysing HRQoL data, it is important to not only consider the statistical significance of effects but also their clinical relevance. This is usually achieved using the concept of minimal clinically important difference, which describes whether or not observed changes are meaningful to patients [22]. Although we found several significant predictors of HRQoL, the effects of all single predictors were below the minimal clinically important difference, if defined as a half standard deviation [23]. Applied to the present study, this would be equivalent to changes of 0.09 for the utilities and of 8.74 for the VAS scores, respectively. However, many patients in our sample were affected by several predictors of reduced HRQoL. Such combinations may have led to clinically important reductions of HRQoL in some patients, as reflected by distribution of values in the histograms of utilities and VAS scores shown in Fig 3.

Previous studies also reported different patient and disease characteristics to be associated with HRQoL in AF patients [24]. Concerning comorbidities, previous studies mentioned sleep apnoea [24, 25], PAD [25], coronary artery disease [24], diabetes [9], and previous stroke [9] as important predictors, which is in line with our results. Heart failure, mentioned also by previous studies [24, 25], was only significant in our VAS score model but not in the utility model. Interestingly, also hypertension remained significantly associated with HRQoL in both models, although it is often not directly felt by the patients. One additional, interesting observation in our study was the strong impact of recurrent falls. This underlines the importance of risk/history of falls assessment in the decision making on medical therapies for AF patients [26]. This notion is consistent with previous observations. For example, in a survey where 41 member centres of the European Heart Rhythm Association EP Research Network completed a web-based questionnaire on frailty, recurrent falls were mentioned as one of the comorbidities most frequently associated with the frailty syndrome and as one important consideration that influences the choice of anticoagulation drug therapy [27]. To our knowledge, no previous study showed the association of falls and HRQoL in AF patients. Interestingly, in our study, the type of anticoagulation at baseline had no impact on HRQoL even if previous studies indicated lower HRQoL in patients treated with vitamin K antagonists for stroke prevention [28] and for venous thromboembolism [29]. This finding may be explained by the fact that we corrected for several factors which may influence the medical decision on the type of the anticoagulation given.

Immediate symptoms of AF such as palpations, fatigue, and syncope played only a minor role in our multivariable models of HRQoL. However, if we excluded the EHRA score, these symptoms became more important. Nevertheless, we decided to include the EHRA score in the reported models, as it was a relatively strong independent predictor of HRQoL and to efficiently consider the absence of symptoms in a substantial proportion of patients.

When looking at the crude association between EHRA scores and HRQoL measurements, both VAS scores and utilities showed a negative association with EHRA class. Differences between the HRQoL medians for each EHRA class were more pronounced in the VAS score. This consistency between patient-reported HRQoL and the physician-assessed EHRA score supports that the EHRA score provides relevant information on the patient’s condition in the daily clinical praxis. Other studies which evaluated the association between EHRA score and patient-reported HRQoL also found a good agreement between the two measures [25, 30].

Some non-modifiable, demographic factors were also associated with reduced HRQoL. Additionally to higher age, we found that women had significantly lower HRQoL than men, also after multivariable adjustment. The effect size of gender was comparable to that of certain comorbidities in the utility (Table 4) and VAS score (Table 5) models. Lower HRQoL in women was already described in previous studies investigating HRQoL in AF patients [7, 9, 24, 31]. The reasons why women frequently report lower HRQoL are not fully understood. Higher rates of depression in women were discussed previously, as well as a different subjective perception of HRQoL [7]. A recent study by Blum et al. showed lower health perception and a higher symptom burden in women than men suffering from AF [2].

### Strengths and limitations

The strength of the present study is the large number of patients included, and that we could consider a wide variety of possible determinant factors including socioeconomic factors such as education. Given only 0.1% missing baseline EQ-5D questionnaires, the results reflect the study population very well. However, the majority of patients enrolled in this cohort is over 65 years old and the study population may therefore not be representative of the full population of AF patients in Switzerland. This could affect mean HRQoL values. Additionally, the study includes mainly Caucasian patients and the results may not be generalizable to other populations. Although the differentiation between AF types is well defined theoretically, allocation in clinical practice is sometimes difficult and misclassifications may occur. Furthermore, to estimate EQ-5D utilities, we had to use the European value set as no value set for Switzerland is available. The observation of higher average values and more patients reporting perfect HRQoL in the questionnaire-based utility part compared to the VAS part of the EQ-5D is expected. Given the design of the instrument, EQ-5D utilities are only responsive to relatively severe impairments of HRQoL [32]. We did not use a disease-specific questionnaire to measure HRQoL, due to two major advantages of the EQ-5D instrument, namely inter-disease comparability and usability for health economic analysis.

### Conclusion

In conclusion, the results showed that the EHRA score is a good marker of HRQoL in AF patients, and that comorbidities have a greater impact on HRQoL than the type of AF. The study provides some relevant details on the multifactorial character of HRQoL in AF patients. This is important not only for better patient assessment in clinical practice, but also for further studies addressing HRQoL in AF patients, including health economic evaluations. Specifically, our findings provide a starting point for further, longitudinal studies of the development of HRQoL of the Swiss-AF patient cohort, where follow-up data continue to be collected. From a health-economic perspective, improving HRQoL may stimulate a decrease in the need for active health care, which may in turn decrease the financial burden of public healthcare, thus contributing to improve and keep up high-quality treatment of AF.

## Supporting information

S1 Table Comorbidities according to AF type. TIA, transient ischemic attack; DVT, deep vein thrombosis; OSAS, obstructive sleep apnoea syndrome; PVI, pulmonary vein isolation; PAD, peripheral artery disease.
(DOCX)

S2 Table Multivariable regression analysis including interaction terms: Predictors of utility in AF patients. Joint p values: age p<0.001, EHRA Score p = 0.001, Education level p = 0.004, Interaction AF type x sleep apnoea p = 0.049. Study centre was included as a random effect variable in the model. PAD, peripheral artery disease; DVT, deep vein thrombosis; EHRA, European Heart Rhythm Association.
(DOCX)

S3 Table Multivariable regression analysis including interaction terms: Predictors of the VAS score in AF patients. Joint p values: age p<0.001, EHRA Score p<0.001, Education level p = 0.010, Interaction AF type x chest pain p = 0.015, Interaction AF type x PAD p = 0.013. Study centre was included as random effect in the model. PAD, peripheral artery disease; DVT, deep vein thrombosis; EHRA, European Heart Rhythm Association; PVI, pulmonary vein isolation.
(DOCX)

S1 File Swiss-AF investigators. (DOCX)
