# Supplementary material for: Health-related quality of life in patients with atrial fibrillation: The role of symptoms, comorbidities, and the type of atrial fibrillation
Source: PLoS One. 2019 Dec 23;14(12):e0226730. doi: 10.1371/journal.pone.0226730 (PMC6927649; doi:10.1371/journal.pone.0226730)
Supplement: S1 Table — TIA, transient ischemic attack; DVT, deep vein thrombosis; OSAS, obstructive sleep apnoea syndrome; PVI, pulmonary vein isolation; PAD, peripheral artery disease. (DOCX) [file pone.0226730.s001.docx]

**S1 Table. Comorbidities according to AF type.** TIA, transient ischemic attack; DVT, deep vein thrombosis; OSAS, obstructive sleep apnoea syndrome; PVI, pulmonary vein isolation; PAD, peripheral artery disease.

|  |  | **Paroxysmal** | **Persistent** | **Permanent** | **p** |
| --- | --- | --- | --- | --- | --- |
| **N (%)** |  | 1079 (44.7) | 709 (29.4) | 624 (25.9) |  |
| **Comorbidities** |  |  |  |  |  |
| History of myocardial infarction | n/N (%) | 164/1079 (15.2) | 104/709 (14.7) | 121/624 (19.4) | **0.035** |
| Hypertension | n/N (%) | 714/1079 (66.2) | 493/709 (69.5) | 473/624 (75.8) | **<0.001** |
| Diabetes | n/N (%) | 166/1079 (15.4) | 112/709 (15.8) | 132/624 (21.2) | **0.006** |
| History of Stroke | n/N (%) | 147/1079 (13.6) | 79/708 (11.2) | 92/624 (14.7) | 0.132 |
| Previous TIA | n/N (%) | 115/1078 (10.7) | 46/709 (6.5) | 58/624 (9.3) | **0.011** |
| History of heart failure | n/N (%) | 205/1078 (19.0) | 197/709 (27.8) | 224/623 (36.0) | **<0.001** |
| History of pulmonary embolism/DVT | n/N (%) | 78/1079 (7.2) | 75/709 (10.6) | 65/624 (10.4) | **0.020** |
| History of heart valve operation | n/N (%) | 81/1079 (7.5) | 81/709 (11.4) | 95/624 (15.2) | **<0.001** |
| Further embolism/occlusions | n/N (%) | 47/1078 (4.4) | 38/709 (5.4) | 41/624 (6.6) | 0.140 |
| OSAS | n/N (%) | 142/1078 (13.2) | 106/709 (15.0) | 112/624 (17.9) | **0.029** |
| PAD | n/N (%) | 75/1079 (7.0) | 52/709 (7.3) | 66/624 (10.6) | **0.022** |
| Renal disease | n/N (%) | 198/1078 (18.4) | 138/709 (19.5) | 171/623 (27.4) | **<0.001** |
| Malignant disease | n/N (%) | 157/1079 (14.6) | 105/709 (14.8) | 123/624 (19.7) | **0.012** |
| History of major bleeding | n/N (%) | 63/1079 (5.8) | 38/709 (5.4) | 51/624 (8.2) | **0.076** |
| Recurrent falls | n/N (%) | 95/1079 (8.8) | 44/709 (6.2) | 63/624 (10.1) | **0.030** |
| Other severe disease | n/N (%) | 298/1078 (27.6) | 186/709 (26.2) | 161/624 (25.8) | 0.663 |
