# Supplementary material for: Health-related quality of life in patients with atrial fibrillation: The role of symptoms, comorbidities, and the type of atrial fibrillation
Source: PLoS One. 2019 Dec 23;14(12):e0226730. doi: 10.1371/journal.pone.0226730 (PMC6927649; doi:10.1371/journal.pone.0226730)
Supplement: S2 Table — Joint p values: age p<0.001, EHRA Score p = 0.001, Education level p = 0.004, Interaction AF type x sleep apnoea p = 0.049. Study centre was included as a random effect variable in the model. PAD, peripheral artery disease; DVT, deep vein thrombosis; EHRA, European Heart Rhythm Association. (DOCX) [file pone.0226730.s002.docx]

**S2 Table. Multivariable regression analysis including interaction terms: Predictors of utility in AF patients.** Joint p values: age p<0.001, EHRA Score p=0.001, Education level p=0.004, Interaction AF type x sleep apnoea p=0.049. Study centre was included as a random effect variable in the model.

PAD, peripheral artery disease; DVT, deep vein thrombosis; EHRA, European Heart Rhythm Association.

|  | **EQ-5D utility** | | | |  |
| --- | --- | --- | --- | --- | --- |
|  | **Coef.** | **p-value** | **95% CI** | |  |
|  |  |  |  |  |  |
|  |  |  |  |  |  |
| **Age groups (<65 as reference)** |  |  |  |  |  |
| 65-<75 | 0.013 | 0.247 | -0.009 | 0.035 |  |
| 75-<85 | -0.011 | 0.352 | -0.034 | 0.012 |  |
| >=85 | -0.048 | **0.003** | -0.080 | -0.017 |  |
| Female | -0.038 | **<0.001** | -0.054 | -0.022 |  |
| Dizziness | -0.022 | **0.028** | -0.041 | -0.002 |  |
| Chest pain | -0.021 | 0.059 | -0.044 | 0.001 |  |
| Fatigue | -0.017 | 0.073 | -0.036 | 0.002 |  |
| Recurrent falls | -0.066 | **<0.001** | -0.089 | -0.041 |  |
| Malignant disease | -0.021 | **0.022** | -0.039 | -0.003 |  |
| PAD | -0.033 | **0.008** | -0.058 | -0.009 |  |
| Hypertension | -0.024 | **0.001** | -0.039 | -0.009 |  |
| Diabetes | -0.031 | **0.001** | -0.049 | -0.013 |  |
| Heart failure | -0.012 | 0.153 | -0.028 | 0.004 |  |
| Renal insufficiency | -0.027 | **0.002** | -0.044 | -0.009 |  |
| History of pulmonary embolism/DVT | -0.053 | **<0.001** | -0.076 | -0.031 |  |
| History of stroke | -0.018 | 0.070 | -0.037 | 0.001 |  |
| **EHRA Score (1 as reference)** |  |  |  |  |  |
| EHRA Score 2 | -0.014 | 0.073 | -0.029 | 0.001 |  |
| EHRA Score 3 | -0.041 | **0.002** | -0.067 | -0.015 |  |
| EHRA Score 4 | -0.067 | **0.007** | -0.116 | -0.018 |  |
| **Educational level (basic as reference)** |  |  |  |  |  |
| middle | 0.016 | 0.142 | -0.005 | 0.037 |  |
| advanced | 0.034 | **0.003** | 0.012 | 0.057 |  |
|  |  |  |  |  |  |
| **AF type (paroxysmal as reference)** |  |  |  |  |  |
| persistent | 0.000 | 0.995 | -0.017 | 0.017 |  |
| permanent | -0.016 | 0.081 | -0.034 | 0.002 |  |
|  |  |  |  |  |  |
| Sleep apnoea | -0.048 | **0.001** | -0.077 | -0.019 |  |
|  |  |  |  |  |  |
| Persistent AF x Sleep apnoea | 0.054 | **0.016** | 0.010 | 0.098 |  |
| Permanent AF x Sleep apnoea | 0.015 | 0.510 | -0.029 | 0.058 |  |
|  |  |  |  |  |  |
| Constant | 0.932 | <0.001 | 0.892 | 0.971 |  |
|  | | | | |  |
