# Supplementary material for: Health-related quality of life in patients with atrial fibrillation: The role of symptoms, comorbidities, and the type of atrial fibrillation
Source: PLoS One. 2019 Dec 23;14(12):e0226730. doi: 10.1371/journal.pone.0226730 (PMC6927649; doi:10.1371/journal.pone.0226730)
Supplement: S3 Table — Joint p values: age p<0.001, EHRA Score p<0.001, Education level p = 0.010, Interaction AF type x chest pain p = 0.015, Interaction AF type x PAD p = 0.013. Study centre was included as random effect in the model. PAD, peripheral artery disease; DVT, deep vein thrombosis; EHRA, European Heart Rhythm Association; PVI, pulmonary vein isolation. (DOCX) [file pone.0226730.s003.docx]

**S3 Table. Multivariable regression analysis including interaction terms: Predictors of the VAS score in AF patients.** Joint p values: age p<0.001, EHRA Score p<0.001, Education level p=0.010, Interaction AF type x chest pain p=0.015, Interaction AF type x PAD p=0.013. Study centre was included as random effect in the model.

PAD, peripheral artery disease; DVT, deep vein thrombosis; EHRA, European Heart Rhythm Association; PVI, pulmonary vein isolation.

|  | **VAS Score** | | | |  |
| --- | --- | --- | --- | --- | --- |
|  | **Coef.** | **p-value** | **95% CI** | |  |
|  |  |  |  |  |  |
| **Age groups (<65 as reference)** |  |  |  |  |  |
| 65-<75 | 2.055 | 0.064 | -0.122 | 4.232 |  |
| 75-<85 | -0.366 | 0.758 | -2.694 | 1.961 |  |
| >=85 | -2.508 | 0.127 | -5.728 | 0.712 |  |
| Female | -2.537 | **0.002** | -4.124 | -0.950 |  |
| Fatigue | -1.672 | 0.076 | -3.521 | 0.177 |  |
| Recurrent falls | -3.449 | **0.005** | -5.840 | -1.059 |  |
| Malignant disease | -5.277 | **<0.001** | -7.040 | -3.514 |  |
| Sleep apnoea | -2.884 | **0.002** | -4.740 | -1.028 |  |
| Hypertension | -2.136 | **0.004** | -3.596 | -0.675 |  |
| Diabetes | -3.088 | **0.001** | -4.895 | -1.280 |  |
| Heart failure | -3.370 | **<0.001** | -4.968 | -1.773 |  |
| Renal insufficiency | -3.435 | **<0.001** | -5.134 | -1.735 |  |
| History of pulmonary embolism/DVT | -1.919 | 0.095 | -4.172 | 0.335 |  |
| History of myocardial infarction | -3.479 | **<0.001** | -5.299 | -1.659 |  |
| History of stroke | -3.785 | **<0.001** | -5.708 | -1.862 |  |
| AF/flutter at study visit | -1.708 | **0.054** | -3.442 | 0.026 |  |
| Previous PVI | 1.738 | **0.055** | -0.039 | 3.516 |  |
| **EHRA Score (1 as reference)** |  |  |  |  |  |
| EHRA Score 2 | -2.624 | **0.001** | -4.108 | -1.140 |  |
| EHRA Score 3 | -6.609 | **<0.001** | -9.174 | -4.046 |  |
| EHRA Score 4 | -4.853 | 0.050 | -9.702 | -0.003 |  |
| **Educational level (basic as reference)** |  |  |  |  |  |
| middle | 3.174 | **0.003** | 1.046 | 5.303 |  |
| advanced | 3.231 | **0.005** | 0.989 | 5.474 |  |
|  |  |  |  |  |  |
| **AF type (paroxysmal as reference)** |  |  |  |  |  |
| persistent | 0.197 | 0.823 | -1.523 | 1.916 |  |
| permanent | -1.484 | 0.185 | -3.679 | 0.711 |  |
|  |  |  |  |  |  |
| Chest pain | -2.044 | 0.175 | -4.999 | 0.910 |  |
|  |  |  |  |  |  |
| Persistent AF x Chest pain | -7.876 | **0.005** | -13.331 | -2.419 |  |
| Permanent AF x Chest pain | -0.638 | 0.818 | -6.073 | 4.798 |  |
|  |  |  |  |  |  |
| PAD | -4.864 | **0.013** | -8.701 | -1.027 |  |
|  |  |  |  |  |  |
| Persistent AF x PAD | -1.894 | 0.534 | -7.862 | 4.073 |  |
| Permanent AF x PAD | 6.637 | **0.020** | 1.060 | 12.213 |  |
|  |  |  |  |  |  |
| Constant | 82.013 | <0.001 | 78.081 | 85.945 |  |
