# Supplementary material for: Health-related quality of life in patients with atrial fibrillation: The role of symptoms, comorbidities, and the type of atrial fibrillation
Source: PLoS One. 2019 Dec 23;14(12):e0226730. doi: 10.1371/journal.pone.0226730 (PMC6927649; doi:10.1371/journal.pone.0226730)
Supplement: S1 File — (DOCX) [file pone.0226730.s004.docx]

**S4 File. Swiss-AF investigators**

University Hospital Basel/Basel University: Stefanie Aeschbacher, Steffen Blum, Leo Bonati, Lorin Fröhlich, Rebecca Gugganig, Thomas Kofler, Philipp Krisai, Christine Meyer-Zürn, Pascal Meyre, Andreas U. Monsch, Christian Müller, Christiane Pudenz, Philipp Reddiess, Javier Ruperti Repilado, Aleksandra Schweizer, Anne Springer, Fabienne Steiner, Samuel Stempfel, Christian Sticherling, Thomas Szucs, Jan van der Stouwe, Gian Voellmin, Leon Zwimpfer. Local Principal Investigator: Michael Kühne; Principal Investigators; Stefan Osswald, David Conen

University Hospital Bern: Faculty: Drahomir Aujesky, Urs Fischer, Juerg Fuhrer, Laurent Roten, Simon Jung, Heinrich Mattle; Research fellows: Luise Adam, Carole Elodie Aubert, Martin Feller, Claudio Schneider, Axel Loewe, Elisavet Moutzouri; Study nurses: Tanja Flückiger, Cindy Groen, Nathalie Schwab. Local Principal Investigator: Nicolas Rodondi

Stadtspital Triemli Zurich: Christopher Beynon, Roger Dillier, Franz Eberli, Simone Fontana, Christine Franzini, Isabel Juchli, Claudia Liedtke, Jacqueline Nadler, Thayze Obst, Noreen Tynan, Xiaoye Schneider, Katrin Studerus, Dominik Weishaupt. Local Principal Investigator: Andreas Müller

Kantonspital Baden: Silke Kuest, Karin Scheuch, Denise Hischier, Nicole Bonetti, Corina Bello, Henriette Isberg, Alexandra Grau, Jonas Villinger, Mary-Monica Papaux, Eva Laube, Philipp Baumgartner, Mark Filipovic, Marcel Frick, Stefanie Leuenberger. Local Principal Investigator: Jürg H. Beer

Cardiocentro Lugano: Angelo Auricchio, Adriana Anesini, Cristina Camporini, Giulio Conte, Maria Luce Caputo, Francois Regoli, Tiziano Moccetti. Local Principal Investigator: Tiziano Moccetti

Kantonsspital St. Gallen: Roman Brenner, David Altmann, Manuela Forrer, Michaela Gemperle. Local Principal Investigator: Peter Ammann

Hôpital Cantonal Fribourg: Mathieu Firmann, Sandrine Foucras. Local Principal Investigator: Daniel Hayoz

Luzerner Kantonsspital: Benjamin Berte, Andrea Kaeppeli, Myriam Roth, Brigitta Mehmann, Markus Pfeiffer, Ian Russi, Kai Schmidt, Vanessa Weberndoerfer, Mabelle Young, Melanie Zbinden; Local Principal Investigator: Richard Kobza

Ente Ospedaliero Cantonale Lugano: Luisa Vicari, Jane Frangi-Kultalahti, Tatiana Terrot. Local Principal Investigator: Giorgio Moschovitis

University Hospital Geneva: Georg Ehret, Hervé Gallet, Elise Guillermet, Francois Lazeyras, Karl-Olof Lovblad, Patrick Perret, Cheryl Teres. Local Principal Investigator: Dipen Shah

University Hospital Lausanne: Nathalie Lauriers, Marie Méan, Sandrine Salzmann. Local Principal Investigator: Jürg Schläpfer

Bürgerspital Solothurn: Nisha Arenja, Andrea Grêt, Sandra Vitelli. Local Principal Investigator: Jan Novak

Ente Ospedaliero Cantonale Bellinzona: Jane Frangi, Augusto Gallino. Local Principal Investigator: Marcello Di Valentino

St. Anna Spital Luzern: Renate Schoenenberger-Berzins.

University of Zurich/University Hospital Zurich: Fabienne Witassek, Matthias Schwenkglenks, Christoph Stippich

Medical Image Analysis Center AG Basel: Ernst-Wilhelm Radue, Tim Sinnecker, Jens Würfel

Clinical Trial Unit Basel: Pascal Benkert, Thomas Fabbro, Patrick Simon, Michael Coslovsky

Schiller AG Baar: Ramun Schmid.
